# *Amaranthus caudatus* Stimulates Insulin Secretion in Goto-Kakizaki Rats, a Model of Diabetes Mellitus Type 2

**DOI:** 10.3390/nu10010094

**Published:** 2018-01-15

**Authors:** Silvia Zambrana, Lena C. E. Lundqvist, Virginia Veliz, Sergiu-Bogdan Catrina, Eduardo Gonzales, Claes-Göran Östenson

**Affiliations:** 1 Instituto de Investigaciones Farmaco Bioquimicas, Universidad Mayor de San Andres, Avenida Saavedra 2224, La Paz 2314, Bolivia; silvia.zambrana@ki.se (S.Z.); viky9287@gmail.com (V.V.); eduardo.gonzales@gmail.com (E.G.); 2 Department of Molecular Medicine and Surgery, Karolinska Institutet, Karolinska University Hospital, 171 76 Stockholm, Sweden; Sergiu-Bogdan.Catrina@ki.se; 3 Department of Molecular Sciences, Swedish University of Agricultural Sciences, P.O. Box 7015, 750 07 Uppsala, Sweden; lena.lundqvist@slu.se; 4 Department of Endocrinology, Metabolism and Diabetes, Karolinska University Hospital, 141 86 Stockholm, Sweden; 5 Centrum for Diabetes, Academic Specialist Centrum, 141 86 Stockholm, Sweden

**Keywords:** *Amaranthus caudatus*, nutraceutical, natural product, diabetes mellitus type 2 diabetes, insulin secretion, Goto-Kakizaki rats

## Abstract

Diabetes Mellitus Type 2 prevalence is increasing worldwide; thus efforts to develop novel therapeutic strategies are required. *Amaranthus caudatus* (*AC*) is a pseudo-cereal with reported anti-diabetic effects that is usually consumed in food preparations in Bolivia. This study evaluated the anti-diabetic nutraceutical property of an *AC* hydroethanolic extract that contains mainly sugars and traces of polyphenols and amino acids (as shown by nalysis with liquid chromatography-mass spectrometry (LC-MS) and nuclear magnetic resonance (NMR)), in type 2 diabetic Goto-Kakizaki (GK) rats and healthy Wistar (W) rats. A single oral administration of *AC* extract (2000 mg/kg body weight) improved glucose tolerance during Oral Glucose Tolerance Tests (OGTT) in both GK rats and in W rats. Long-term treatment (21 days) with *AC* (1000 mg/kg b.w.) improved the glucose tolerance evaluated by the area under the curve (AUC) of glucose levels during the OGTT, in both GK and W rats. The HbA1c levels were reduced in both GK (19.83%) and W rats (10.7%). This effect was secondary to an increase in serum insulin levels in both GK and W rats and confirmed in pancreatic islets, isolated from treated animals, where the chronic *AC* exposure increased the insulin production 4.1-fold in GK and 3.7-fold in W rat islets. Furthermore, the effect of *AC* on in vitro glucose-dependent insulin secretion (16.7 mM glucose) was concentration-dependent up to 50 mg/mL, with 8.5-fold increase in GK and 5.7-fold in W rat islets, and the insulin secretion in perifused GK and W rat islets increased 31 and nine times, respectively. The mechanism of action of *AC* on insulin secretion was shown to involve calcium, PKA and PKC activation, and G-protein coupled-exocytosis since the *AC* effect was reduced 38% by nifedipine (L-type channel inhibitor), 77% by H89 (PKA inhibitor), 79% by Calphostine-C (PKC inhibitor) and 20% by pertussis toxin (G-protein suppressor).

## 1. Introduction

Diabetes Mellitus Type 2 (DMT2) is a metabolic disease characterized by chronically elevated levels of blood glucose, due to impaired insulin secretion and insulin resistance [1,2]. The prevalence of diabetes has been increasing worldwide with the most dramatic increase in low-income countries [3]. According to the International Diabetes Federation (IDF), the number of people with diabetes is predicted to increase to 642 million by 2040 [4]. Moreover, diabetic patients are at higher risk of morbidity and mortality due to the development of diabetes complications [5,6]. Despite the present availability of several anti-diabetic drugs, development of novel therapeutic strategies with low adverse effects and better adherence is needed [7]. Adverse effects, such as hypoglycemias and weight gain, can impair glucose control and reduce treatment compliance.

Natural products are potential sources of novel therapies, which with a good safety profile have become an attractive complement to the regular pharmacological therapies [8]. A number of natural products have been reported to have anti-diabetic effects [9,10,11,12], such as α-lipoic acid [9,13], strawberry extracts [14], flavonoids [11]. Thus, the approach of this study is the search for anti-diabetic products using food plants, which may have medicinal properties (nutraceuticals) [15,16]. A nutraceutical is a food or part of a food that has a beneficial pharmaceutical benefit beyond its nutritional value [17].

Based on the use of Bolivian traditional foods, we evaluated the anti-diabetic property of *Amaranthus caudatus* (*AC*), traditional name kiwicha. *AC* is a gluten-free pseudocereal [18,19], native of the Bolivian valley region, and its seeds are traditionally consumed in beverages, food preparations such as soups, bread, or as toasted flour (pito). *AC* is an attractive nutraceutical crop due to its high protein content (rich in lysine), dietary fiber, and bioactive compounds such as tocopherols, phenolic compounds, folate [20,21] squalene, phytates, and vitamins [22]. Among its anti-diabetic effects, *AC* seed water decoction extract [23] and methanolic extract [24] showed α-amylase inhibitory activity. The methanol extract of *AC* leaves reduced blood glucose level and improved the lipid profile of rats with streptozotocin-induced diabetes [25].

In the present study, the *AC* anti-diabetic effect was tested in type 2 diabetic Goto-Kakizaki rats (GK) and in healthy Wistar rats (W). The effect on insulin release was evaluated in pancreatic islets and to explore the in vitro mechanism of action, and inhibitory compounds of insulin secretion pathway were used in batch incubation experiments.

## 2. Materials and Methods

### 2.1. Animals

Male healthy Wistar rats (W) and spontaneously type 2 diabetic GK (Goto-Kakizaki) rats (150–300 g) were used in this study. GK rats, originally derived from glucose intolerant W rats, were bred in the animal facilities of the department of Molecular Medicine and Surgery, KI [26], while W rats were purchased from a commercial breeder (Charles River, Sweden). Experiments were done after one week of adaptation in the animal facilities. Animals were kept at 22 °C with alternating 12 h light- dark cycle and free access to food and fresh water. The study was approved by the Laboratory Animal Ethics Committee of the Karolinska Institutet (approval Dnr N50/2014).

### 2.2. Plant Material

The plant material was collected from local producers in Tomina municipality, Tomina Province, Chuquisaca (latitude 19°25′53.96′′ S and longitude 64°15′5.44′′ W). One voucher specimen (No. EG-1, Amaranthaceae) was identified and certified by the Herbario Nacional de Bolivia from Universidad Mayor de San Andrés and has been deposited at the Department of Pharmacology at the Instituto de Investigaciones Farmaco Bioquimicas, UMSA, La Paz, Bolivia.

### 2.3. Plant Extract Preparation

The *AC* hydro-ethanolic extract was prepared with 200 g of powdered seeds macerated for 48 h with 250 mL of 70% ethanol. The maceration procedure was repeated five times to maximize the extraction yield. Ethanol solvent was evaporated using a rotary evaporator (Heidolph, Schwabach, Germany) and the water fraction was dried under pressure in a freeze dryer (Labconco, Kansas City, MO, USA). Crude extracts obtained had an appearance of a light powder with a yield of 6.5% *w*/*w*. For experiments, extracts were dissolved in distillated water and stock solutions were sterilized using 0.2 μm Millipore filter (Sigma-Aldrich, St. Louis, MO, USA).

### 2.4. LC-MS (Liquid Chromatography-Mass Spectrometry) Analysis

The *AC* extract was dissolved in Milli-Q (Millipore S.A.S., Molsheim, France) water and filtrated through a 0.22 µm filter before performing HPLC-HRMS (High Performance Liquid Chromatography—High Resolution Mass Spectrometry analysis), without any further purification. HPLC analysis was performed using an Agilent 1100 system (Agilent Technologies, Palo Alto, CA, USA) equipped with a Discovery 150 × 4.6 mm reversed phase C18 column (Sigma-Aldrich Supelco, Bellefonte, PA, USA). The mobile phase was composed of water with 0.1% formic acid (A), and acetonitrile (B). A stepwise gradient was used starting with 95% (A): 5% (B), and held there for 5 min, then changed to 80% (B) in 40 min, and finally return to initial conditions 95% (A): 5% (B) in 45 min, with a flow of 0.8 mL/min. For the HRMS detection, a Bruker’s MaXis Impact ESI Q-TOF mass spectrometer (Bruker Daltonics GmbH, Bremen, Germany) with sodium formate (positive) as calibrant (positive scanning mode, *m*/*z* 50–1500) was used. Ultra-violet (UV) detection was done using an Agilent 1100 series Diode Array Detector (DAD).

The following chemicals were used as standards: Caffeic acid (Sigma-Aldrich, St. Louis, MO, USA); *p*-coumaric acid (Koch-light Laboratories Ltd., Cardiff, UK); ferulic acid (Fluka AG, Buch/SG, Switzerland); vanillic acid (Merck, Kenilworth, NJ, USA); kaempferol (Sigma-Aldrich, St. Louis, MO, USA); myricetin (Sigma-Aldrich, St. Louis, MO, USA); rutin (Sigma-Aldrich, St. Louis, MO, USA); squalene (Fluka AG, Buch/SG, Switzerland); quercetin (Sigma-Aldrich, St. Louis, MO, USA); inositol (Pfanstiehl Chemicals Co., Waukegan, IL, USA); fructose (Pfanstiehl Chemicals Co. Waukegan, IL, USA); glucose (Nutritional Biochemicals Co., Cleveland, OH, USA); maltose (Sigma-Aldrich, St. Louis, MO, USA); raffinose (Kebo, Stockholm, Sweden); isoleucine (Sigma-Aldrich, St. Louis, MO, USA); leucine (Merck, Kenilworth, NJ, USA); phenylalanine (Merck, Kenilworth, NJ, USA); tryptophan (Merck, Kenilworth, NJ, USA); tyrosine (Merck, Kenilworth, NJ, USA).

### 2.5. NMR Analysis

The *AC* extract was dissolved in Milli-Q water and filtrated through a 0.22 µm filter before being lyophilized, re-dissolved in D2O and submitted to NMR analysis. The NMR data were recorded at 25 °C with a Bruker AVANCE™ III (Bruker BioSpin GmbH, Rheinstetten, Germany) 600 MHz spectrometer equipped with a 5 mm 1H/13C/15N/31P inverse detection QXI probe, with a z-gradient. The ^13^C and ^1^H chemical shifts were measured using acetone as an internal standard (δ = 2.225 and 31.05 ppm for proton and carbon respectively). The data were acquired and processed using Bruker software TopSpin 3.1. (Bruker BioSpin GmbH, Rheinstetten, Germany). The ^1^H–^13^C HSQC and TOCSY spectra were recorded using standard pulse sequences from the Bruker library. A mixing time of 120 ms was used for the TOCSY experiment.

### 2.6. Sub-Acute Oral Toxicity

The *AC* extract was evaluated for its potential sub-acute oral toxicity, for 28 days, according to the guidelines set by the Organization for Economic Cooperation and Development (OECD) guideline 407 [27]. Briefly, W rats received the *AC* extract added to the regular chow food in a quantity to reach a daily dose of 1000 mg/kg b.w. During the treatment, changes in skin, fur, eyes, the occurrence of secretions, lacrimation, and piloerection were monitored. At the end point, blood samples were collected to determine hematological and serum biochemical parameters. Body weights did not differ between the groups (Supplementary Materials Figure S1).

### 2.7. Oral Glucose Tolerance Test (OGTT)

*AC* hydroethanolic extract (2000, 1000 and 500 mg/kg b.w.) was administrated orally to 10–12 h fasted GK and W rats (*n* = 6 per group), one hour before the OGTT. The evaluation started with an oral glucose challenge of 2 g/kg b.w. for GK rats and 3 g/kg b.w. for W rats. Blood samples were collected, from the tip of the tail, immediately after the glucose administration (time 0), 30, 60, 90, and 120 min [28]. To measure glycemia, a glucometer Accu-check Aviva (Roche Diagnostic GmbH, Indianapolis, IN, USA) was used. Serum insulin levels were measured at time 0 and 30 min by a radioimmunoassay (RIA) [29]. The placebo group received vehicle (distilled water).

### 2.8. Long-Term Treatment Evaluation

Long-term oral *AC* treatment with a daily dose during 21 days was evaluated in GK and W rats (*n* = 6 per group). Animals were grouped as follow: group 1: GK rats treated with 1000 mg/kg b.w. of *AC*; group 2: GK rats, treated with vehicle, distilled water; group 3: W rats treated with 1000 mg/kg b.w. of *AC*; group 4: W rats treated with vehicle, distilled water. Body weights (Supplementary Materials Figure S2) and non-fasting glucose levels were measured every third day, and there were no significant differences in body weights between the groups. The OGTT was performed on days 0, 10, and 20; blood samples were collected to measure serum insulin by RIA and glycated hemoglobin (HbA1c) by ELISA (Crystal Chem INC, Elk Grove Village, IL, USA). At the end point, day 21, pancreatic tissue was collected to isolate pancreatic islets to evaluate the insulin secretion.

### 2.9. Pancreatic Islets Isolation

Pancreatic islets were isolated using collagenase type 1 (Sigma-Aldrich, St. Louis, MO, USA) dissolved in 10 mL of Hank’s Balanced Solution (HBSS) (Sigma-Aldrich, St. Louis, MO, USA). Altogether 9 mg collagenase for W and 24 mg collagenase for GK rats, were injected through the bile duct to insufflate the pancreas tissue [30,31]. The tissue was transferred to a test tube that was incubated in a water bath without shaking for 24 min at 37 °C, then collagenase was washed away with HBSS by centrifugation, and digested tissue was filtrated trough a restrainer. Finally, islets were separated from exocrine pancreatic tissue by centrifugation using a mixture of Histopaque 1119 and 1077 (Sigma-Aldrich, St. Louis, MO, USA). Islets were hand-picked using micro pipettes under a stereomicroscope and then cultured overnight at 37 °C, in an atmosphere of 5% CO_2_–95% air, in RPMI 1640 medium (SVA, Stockholm, Sweden), supplemented with 30 mg l-glutamine (Sigma-Aldrich, St. Louis, MO, USA), 11 mM glucose (Sigma-Aldrich, St. Louis, MO, USA), antibiotics (100 IU/mL penicillin and 0.1 mg/mL streptomycin) (Invitrogen, CA, USA) and heat-inactivated fetal calf serum (10%) (Sigma-Aldrich, St. Louis, MO, USA).

### 2.10. Islet Insulin Secretion

Overnight cultured islets were pre-incubated 30–45 min at 37 °C and 3.3 mM glucose in Krebs-Ringer bicarbonate (KRB) buffer (NaCl 118.4 mM, KCl 4.7 mM, MgSO_4_ 1.2 mM, KH_2_PO_4_ 1.2 mM, CaCl_2_ 1.9 mM, NaHCO_3_ 25 mM, 4-(2-hydroxyethyl)-1-piperazineethanesulfonic acid (HEPES) 10 mM and 0.2% bovine serum albumin) (Sigma-Aldrich, St. Louis, MO, USA). Then, batches of three islets of similar size were incubated for 60 min in 300 μL of KRB at 3.3 mM or 16.7 mM glucose, with or without *AC* extract, at 37 °C in a water bath with gentle shaking [28,31]. After incubation, 200 μL of incubation media were collected to new tubes and kept frozen at −20 °C prior to determination of insulin by radioimmunoassay (RIA).

### 2.11. Islet Perifusion

To explore the effect of *AC* on kinetics of insulin release, batches of 40 or 50 isolated W and GK rat islets were layered between polystyrene beads (Bio-Rad Laboratories, Inc., Hercules, CA, USA) in a perifusion chamber, and KRB buffer was perifused continuously using a peristaltic pump (Ismatec SA, Zurich, Switzerland). To establish the basal insulin secretion rate, islets were perifused with 3.3 mM glucose in KRB for 20 min (−20 to min 0). The KRB buffer content was then changed to 3.3 mM glucose plus *AC* (20 mg/mL), from time 0 to 14 min; to 16.7 mM glucose plus *AC* (20 mg/mL), from time 16 to 30 min; and to 3.3 mM glucose without *AC*, for the last 20 min [28]. Perifusion buffer was collected every 2 min and stored at −20 °C for later insulin determination by RIA. The AUC in presence of *AC* was calculated subtracting the basal value at the beginning of each treatment; for low glucose, time 0 (period 0 to 14 min) and for high glucose, time 16 (period 16 to 30 min) and compared to same periods of untreated islets.

### 2.12. Mechanisms of Insulin Secretion Induced by AC

To study the mechanism of *AC* effect on insulin secretion, GK and W rat islets were incubated in presence of different compounds that interfere with different steps of the insulin secretion pathway. The effect on the adenosine triphosphate (ATP)-sensitive potassium channels (K-ATP) was evaluated using 0.25 mM diazoxide (DX), an opener of K-ATP channels (Sigma-Aldrich, St. Louis, MO, USA). Islets were incubated in 3.3 or 16.7 mM glucose with *AC* (20 mg/mL) and DX alone or with DX and 50 mM of KCl (to depolarize the β-cells) [32]. The effect on Ca^2+^ channels was evaluated using 10 μM nifedipine (NF) (Sigma-Aldrich, St. Louis, MO, USA), an inhibitor of L-type Ca^2+^ channels. Islets were incubated in 3.3 or 16.7 mM glucose KRB with *AC* (20 mg/mL) plus NF [32]. To evaluate the role of protein kinase A (PKA) and protein kinase C (PKC) on the effect of *AC*, islets were incubated with *AC* (20 mg/mL) with 10 μM H89 (Sigma-Aldrich, St. Louis, MO, USA), a PKA-inhibitor, or with 1.5 μM calphostin-C (Cal-C) (Sigma-Aldrich, St. Louis, MO, USA), a PKC inhibitor, in KRB containing 3.3 mM and 16.7 mM glucose [31,32]. Finally, to explore the role of G-protein-coupled exocytosis, a G-protein suppressor, pertussis toxin (PTx) (Sigma-Aldrich, St. Louis, MO, USA) was used. Islets were pretreated at 37 °C overnight with 100 ng/mL PTx in complete RPMI 1640 culture medium (with additions as given above). After exposure, islets were incubated with 20 mg/mL of *AC* in 3.3 mM or 16.7 mM glucose KRB [32,33]. For all the treatments, 200 μL aliquots of KRB medium were collected and stored at −20 °C for insulin determination by RIA.

### 2.13. Cytotoxicity

Cellular toxicity was evaluated in batches of W islets exposed to *AC* extract (5–50 mg/mL) in complete RPMI 1640 culture medium (Sigma-Aldrich, St. Louis, MO, USA) during 2 and 24 h at 37 °C. After treatment, cell viability was determined by MTT assay (Sigma-Aldrich, St. Louis, MO, USA) [34].

### 2.14. Statistical Analysis

Results are presented as mean ± SEM. Statistical differences between groups were analyzed using two-way analysis of variance (ANOVA) for OGTT, serum insulin, glycated hemoglobin, insulin secretion and insulin kinetics evaluations whereas paired Student’s t-test was used for AUC analysis. Bonferroni’s Post Hoc Test was used for correction of multiple testing. A *p* value of less than 0.05 was considered significant. Data were analyzed using Prism Graph Pad Software (San Diego, CA, USA).

## 3. Results

### 3.1. Phytochemical Constituents of AC Extract

**Figure 1:**
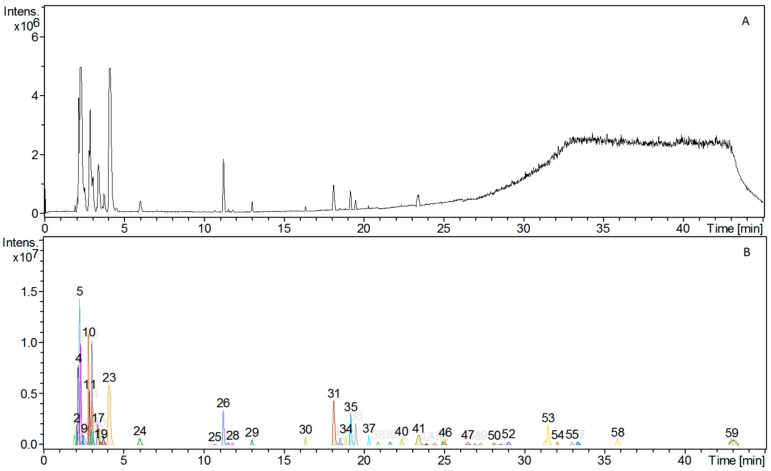
The *Amaranthus caudatus* (*AC*) extract consists of a complex mixture of phytochemicals. A high pressure liquid chromatography (HPLC) chromatogram with (**A**) total ion current (TIC) chromatogram and (**B**) processed chromatogram showing the presence of at least 59 different peaks each corresponding to a compound with a discrete molecular weight. From the retention time and protonated molecular ions [M + H]^+^ from the MS spectra, some peaks corresponding to amino acids were assigned: Peak 18, iso-leucine MW 131; Peak 21, leucine MW 131; Peak 24, phenylalanine MW 165; and Peak 26, tryptophan MW 204.

The HPLC-HRMS analysis of the *AC* extracts showed more than 59 different types of phytochemicals (Figure 1).

**Figure 2:**
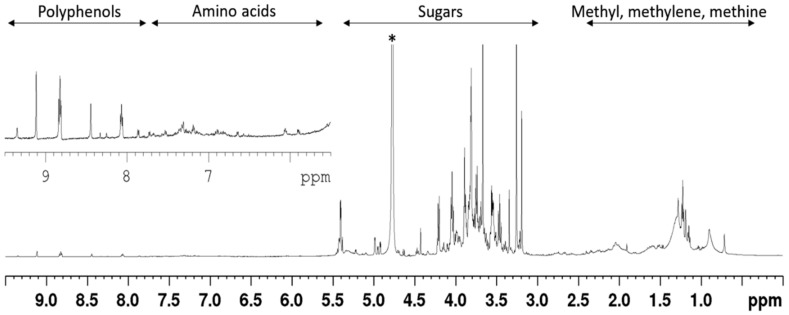
The ^1^H-NMR (Nuclear Magnetic Resonance) spectrum indicates that the *AC* extract consists primarily of sugars, and of minor amount of polyphenols and amino acids. The extract was analysed by NMR spectrometry in D_2_O at 25 °C. The ^1^H-NMR spectrum, indicates the presence of at least four groups of components, with sugars constituting the major fractions of the extract; * denote the solvent (D_2_O) signal.

**Figure 3:**
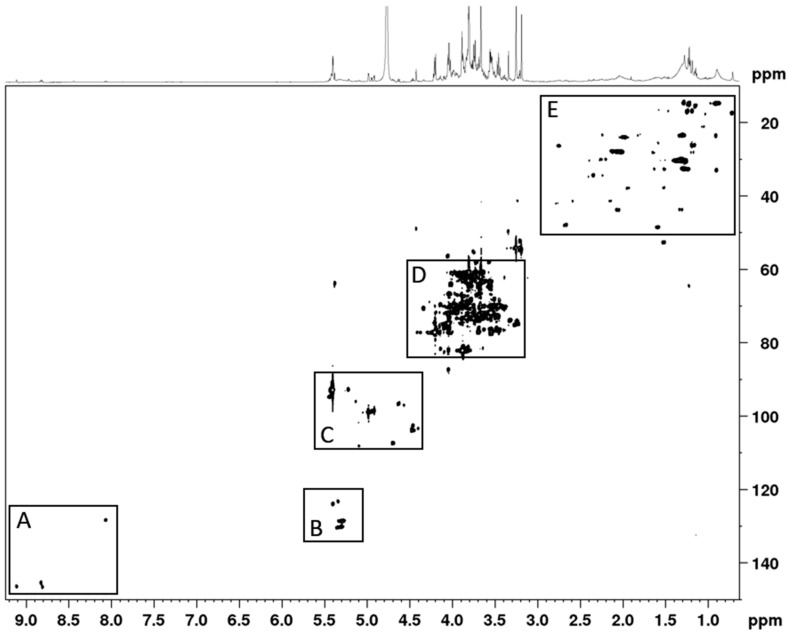
The HSQC (Heteronuclear Single Quantum Coherence) spectrum shows that the *AC* extract consists primarily of sugars, and of minor amount of polyphenols and amino acids. The ^1^H-^13^C-HSQC spectrum shows the C_2_/H_2_ to C_6_/H_6_ sugar ring signals (region D); the anomeric C_1_/H_1_ sugars signals (region C); olefinic signals (region B); aromatic/polyphenol signals (region A); and methyl, methylene, methine signals (region E).

Of the 19 reference standards, only four amino acids could unambiguously be assigned, due to the high amount of sugars and co-eluting peaks. The identified amino acids were iso-leucine, leucine, phenylalanine, and tryptophan. Due to the complexity of the chromatogram no further attempt was made to identify the chemical composition of the phytochemicals constituting the extract. Furthermore, NMR data indicated that the extract consisted primarily of sugars (Figure 2 and Figure 3) and that a minor amount of polyphenols and amino acids were also present. When the extract was dissolved as 20 mg/mL, the glucose concentration was 2 mM.

### 3.2. AC Improves Glucose Tolerance in GK and W Rats by Increasing Serum Insulin Levels

**Figure 4:**
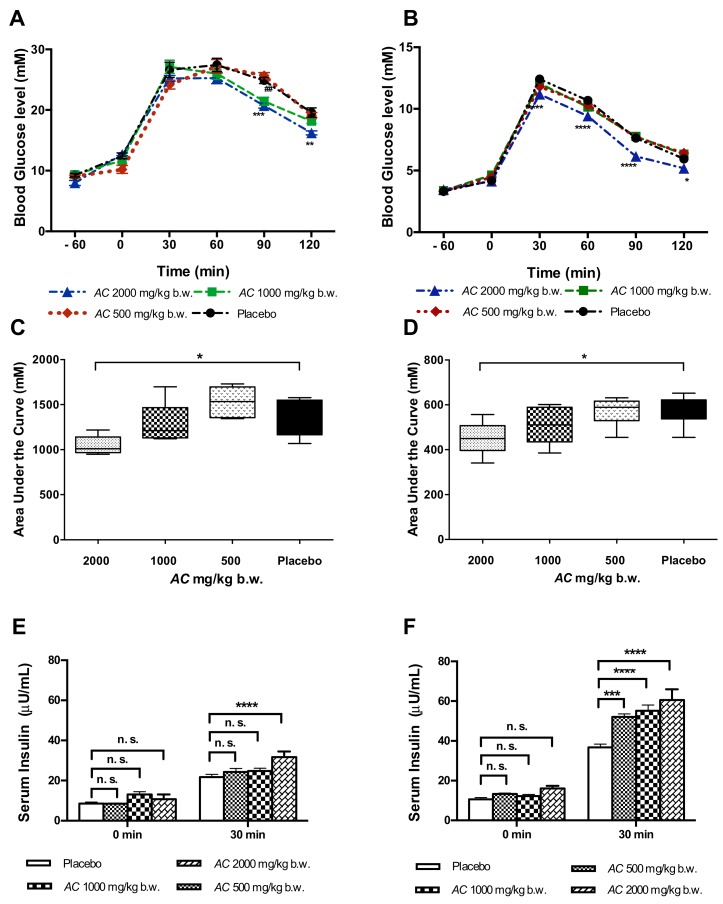
*AC* improves glucose tolerance and increases serum insulin levels. *AC* (500–2000 mg/kg b.w.) effect was evaluated during the OGTT in 12 h-fasted, animals received a single oral administration of *AC* extract one hour before glucose-challenge and glycemia was determined at 0, 30, 60, 90, and 120 min; GK rats (**A**) and W rats (**B**); The AUC of glucose was calculated from time 0 to 120 min in GK rats (**C**) and W rats (**D**); The effect of *AC* on serum insulin during the OGTT in GK rats (**E**) and W rats (**F**) was determined from 0–30 min. Data are presented as means ± SEM (*n* = 6). * *p* < 0.05, ** *p* < 0.01, *** *p* < 0.001, **** *p* < 0.0001 when compared to the placebo group; ## *p* < 0.01.

*AC* (2000 mg/kg b.w.) improved the glucose tolerance during the OGTT in GK rats, at 90 min (20.7 ± 0.4 mM) (*p* < 0.001) and at 120 min (16.2 ± 0.3 mM) (*p* < 0.01) after glucose administration as compared to placebo treated GK rats (90 min, 24.9 ± 0.5 mM; 120 min 19.5 ± 0.7 mM) (Figure 4A). *AC* in a lower dose (1000 mg/kg b.w.) improved glucose tolerance only at 90 min (21.4 ± 0.6 mM) (*p* < 0.01). No effect was found with the lowest dose of *AC* tested (500 mg/mL). In W rats, the *AC* effect on glucose tolerance was observed only at the highest dose tested (2000 mg/kg b.w.) starting 30 min after glucose challenge (11.2 ± 0.2 mM) (*p* < 0.001) and during the following time points 60 min (9.4 ± 0.1 mM) (*p* < 0.0001), 90 min (6.1 ± 0.1 mM) (*p* < 0.0001) and 120 min (5.2 ± 0.1 mM) (*p* < 0.05) (Figure 4B).

The *AC* effect during the OGTT was also estimated by the calculation of the AUC of glucose. In GK rats treated with *AC* (2000 mg/kg b.w.) the AUC of glucose was significantly reduced to 1044.6 ± 47.1 mM/120 min compared with the GK rats exposed to placebo (1379.7 ± 132.5 mM/120 min; *p* < 0.05) (Figure 4C). Similarly, in W rats *AC* treatment at the highest dose (2000 mg/kg b.w.) reduced the AUC of glucose (450.5 ± 30.3 mM/120 min) compared to placebo-treated W rats (752.0 ± 27.0 mM/120min; *p* < 0.05) (Figure 4D).

During the OGTT, *AC* was able to increase serum insulin levels during the first 30 min after glucose challenge. In GK rats, *AC* (2000 mg/kg b.w.) increased insulin levels up to 31.7 ± 2.8 μU/mL compared to placebo (21.7 ± 1.4 μU/mL) (*p* < 0.0001) (Figure 4E). In W rats, *AC* also increased serum insulin levels at all *AC* doses tested 500 mg/kg b.w. (52.0 ± 1.5 μU/mL), 1000 mg/kg b.w. (55.1 ± 2.8 μU/mL) and 2000 mg/kg b.w. (60.6 ± 5.4 μU/mL) compared to placebo (36.8 ± 1.6 μU/mL) (*p* < 0.0001) (Figure 4F).

### 3.3. Long-Term Oral Treatment with AC Improves Glucose Tolerance and Insulin Secretion

**Figure 5:**
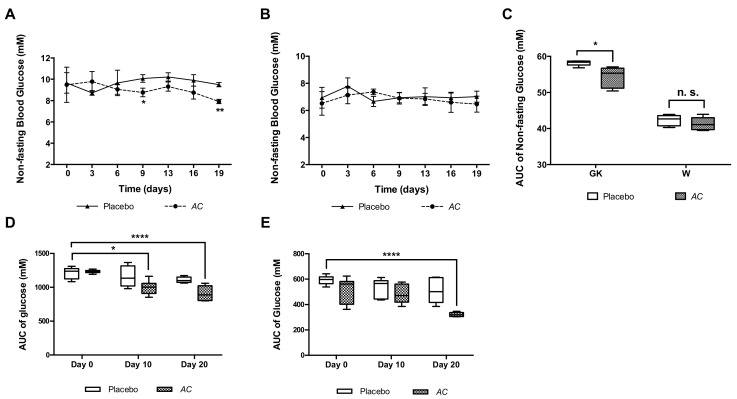
*AC* oral long-term treatment reduces the non-fasting glucose and improves glucose tolerance. The non-fasting glucose was determined every third day of treatment in GK (**A**) and W rats (**B**); the AUC of the non-fasting glucose were calculated from the interval 0–19 days (**C**); The AUC of glucose was calculated for each OGTT performed (day 0, 10 and 20) in GK (**D**) and W rats (**E**). Data are presented as means ± SEM (*n* = 6). * *p* < 0.05, ** *p* < 0.01, **** *p* < 0.0001 when compared to Placebo.

The long-term (21 days) oral treatment with *AC* (1000 mg/kg b.w.) had a non-significant tendency to reduce the non-fasting glucose in GK rats at a few time points, measured every third day during the treatment (Figure 5A). No significant reduction was observed in W rats (Figure 5B). However, when the AUC of non-fasting glucose was calculated, a significant difference was observed between *AC*-treated GK rats (54.4 ± 1.2 mM/19 days) and placebo-treated GK rats (58.2 ± 0.3 mM/19 days) (*p* < 0.05) (Figure 5C).

The glucose tolerance was improved by *AC* treatment reflected by the AUC of the serum glucose during OGTT in GK rats at day 10 (996.5 ± 42.5 mM/120 min) (*p* < 0.05) and day 20 (910.0 ± 47.3 mM/120 min) (*p* < 0.0001) compared to placebo GK rats at day zero (1209.0 ± 39.0 mM/120 min) (Figure 5D). In W rats *AC* reduced the AUC only at day 20 (321.3 ± 6.8 mM/120 min) compared to placebo W rats at day zero (593.3 ± 14.6 mM/120 min) (*p* < 0.0001) (Figure 5E).

The effect of *AC* on glucose tolerance was also detected by analyzing differences between each time point along the OGTT. In GK rats, at day 10 and 20 compared to day zero of treatment, during the intervals of 60–120 min of the OGTT (*p* < 0.01–*p* < 0.0001) and during the interval 90–120 min of the OGTT (*p* < 0.05–*p* < 0.0001), when compared to placebo-treated GK rats (Supplementary Materials Table S1). Similarly, in W rats where *AC* improved glucose tolerance at day 10 (90 and 120 min of the OGTT) and day 20 (30–120 min of the OGTT), compared to day zero of treatment (*p* < 0.05–*p* < 0.0001) and at day 20 (30–120 min of the OGTT) (*p* < 0.05–*p* < 0.0001), when compared to placebo-treated W rats (Supplementary Materials Table S1).

**Figure 6:**
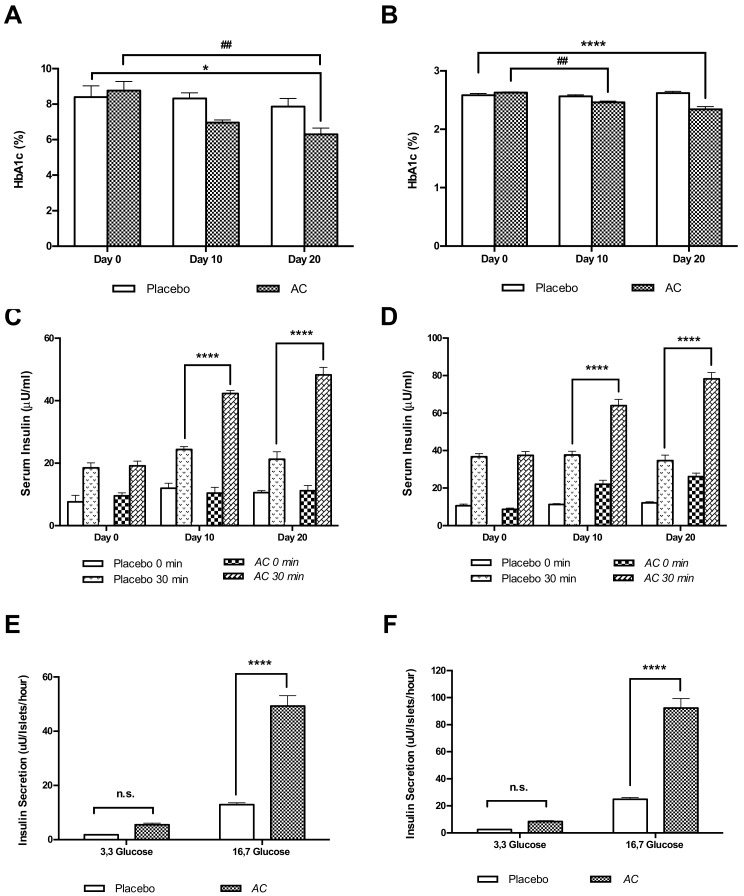
*AC* oral long-term treatment reduces the plasma HbA1c, increases serum insulin, and improved insulin secretion in pancreatic islets of treated animals. The plasma HbA1c were measured during each OGTT in GK rats (**A**) and in W rats (**B**); Serun Insulin were measured during each OGTT in GK rats (**C**) and in W rats (**D**) Data are presented as means ± SEM (*n* = 6). Pancreatic islets were isolated at the end point of treatment (day 21) from GK rats (**E**) and W rats (**F**) were cultured at low (3.3 mM) and high (16.7 mM) glucose. Data are presented as means ± SEM (*n* = 4). Insulin concentration was measured by RIA. * *p* < 0.05, **** *p* < 0.0001 when compared to placebo group; ## *p* < 0.01, when compared to values from the same group.

The plasma HbA1c levels were reduced by 19.8% in GK rats treated with *AC* at day 20 (6.3 ± 0.35%) compared to placebo (7.8 ± 0.46%) (*p* < 0.05) and by 27.6% compared to day zero (8.7 ± 0.50%) (*p* < 0.01) (Figure 6A). In W rats HbA1c levels were reduced by 2.2% (2.4 ± 0.01) at day 10 and by 10.7% at day 20 (2.3 ± 0.05%) (*p* < 0.0001) and compared to placebo W rats (2.5 ± 0.02, day 10 and 2.6 ± 0,02, day 20) (Figure 6B).

Serum insulin levels in GK rats treated with *AC* were increased during the OGTT 1.7-fold at day 10 (*p* < 0.0001) and 2.3-fold at day 20 (*p* < 0.0001) (Figure 6C), and in W rats 1.6-fold at day 10 (*p* < 0.0001) and 2.2-fold at day 20 (*p* < 0.0001) (Figure 6D). Furthermore, *AC* augmented insulin secretion in pancreatic islets isolated from treated animals. Thus, insulin secretion increased 4.1-fold at high glucose (16.7 mM) in islets isolated from *AC* treated GK rats, compared to the secretion in islets isolated from placebo treated GK rats (*p* < 0.0001) (Figure 6E). Similarly, *AC* treatment in W rats increased islet insulin secretion at 16.7 mM glucose 3.7-fold, compared to the secretion in islets from placebo treated W rats (*p* < 0.0001) (Figure 6F).

The sub-acute toxicity studies did not show significant differences between *AC-*treated W rats and placebo group of the hematological indicators i.e., red and white blood cells number, hematocrit and hemoglobin, and serum biochemical parameters i.e., triglycerides, cholesterol, glucose, creatinine, alkaline phosphatase, aspartate aminotransferase, and alanine transferase (Supplementary Materials Table S2).

### 3.4. AC Stimulates In Vitro Insulin Secretion in a Concentration-Dependent Manner

**Figure 7:**
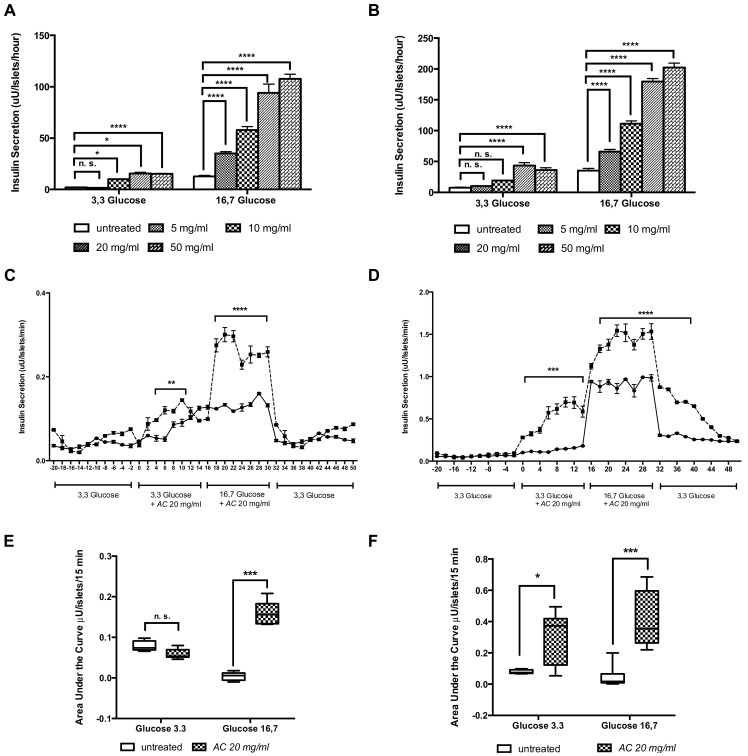
*AC* stimulates the in vitro insulin secretion in pancreatic islets. Insulin secretion was evaluated in GK rat islets (**A**) and W rat islets (**B**) cultured at low (3.3 mM) and high (16.7 mM) glucose in presence of *AC* (5–200 mg/mL). Data are presented as means ± SEM (*n* = 8). Batches of (50) GK (**C**) and Wistar (**D**) rats islets were perifused with 3.3 mM glucose, from time 0 to 14 min, and with 16.7 mM glucose, from time 16 to 30 min, in presence ---■--- or absence —●— of *AC* (20 mg/mL). The AUC of the insulin secretion from the intervals at 3.3 mM and 16.7 mM of glucose in presence or absence of *AC* was calculated for GK (**E**) and W (**F**). rats islets. Data are presented as means ± SEM (*n* = 4). * *p* < 0.05, ** *p* < 0.01, *** *p* < 0.001, **** *p* < 0.0001 when compared to untreated islets.

In GK rat islets, insulin secretion was stimulated by 10, 20 and 50 mg/mL *AC* at 3.3 mM glucose, and by 5, 10, 20 and 50 mg/mL *AC* at 16.7 mM glucose in a concentration-dependent manner, with a maximal 7.9-fold increase at low glucose (3.3 mM) (*p* < 0.0001) and 8.5-fold increase at high glucose (16.7 mM), (*p* < 0.0001) compared to untreated islets (Figure 7A). In W rats islets, the insulin secretion was increased in all concentrations tested up to 50 mg/mL, 5.1-fold (3.3 mM) and 5.7-fold (16.7 mM) compared to untreated islets (Figure 7B). Significant differences were found in lower concentrations until 10 mg/mL (3.3 mM) and 5 mg/mL (16.7 mM) in both GK and W rat islets. No cytotoxic effect was observed in tested concentrations (Supplementary Materials Figure S3).

### 3.5. The AC Effect on Kinetics of Insulin Secretion

To monitor the *AC* effect on the kinetics of insulin secretion, isolated islets were perifused with KRB buffer containing *AC* extract (20 mg/mL). Significant differences were found during the period of 4 to 12 min (*p* < 0.01), when GK rat islets were perfused with *AC* at 3.3 mM glucose and during 16 to 32 min (*p* < 0.0001), when the islets were perfused with *AC* at 16.7 mM glucose, compared to the respective time point from untreated GK rat islets (Figure 7C). Similar pattern was observed in W rat perifused islets during the time period of 0 to 14 min (3.3 mM) (*p* < 0.001) and 18 to 40 min (16.7 mM) (*p* < 0.0001) (Figure 7D).

The effect on insulin secretion was also observed when the AUC of insulin secretion was calculated. In GK islet perifused with 16.7 mM of glucose (14–30 min), *AC* increased insulin secretion 37.2-fold (*p* < 0.001) (Figure 7E) and in W rats with 3.9-fold at 3.3 mM of glucose (0–14 min) (*p* < 0.05) and with 9.2-fold at 16.7 mM of glucose (16–30 min) (*p* < 0.001) (Figure 7F).

### 3.6. AC Stimulates Insulin Secretion through the Activation of PKC and PKA Systems and Partially by L-Type Calcium Channels and G Protein-Coupled Exocytosis

Diazoxide (DX), a selective ATP-sensitive K^+^-channel opener, did not reduce the effect of *AC* on insulin secretion. Not even combined exposure to DX and high concentrations of KCl to induce a transient membrane depolarization altered the *AC* effect, in GK or W rat islets (Supplementary Materials Figure S4).

**Figure 8:**
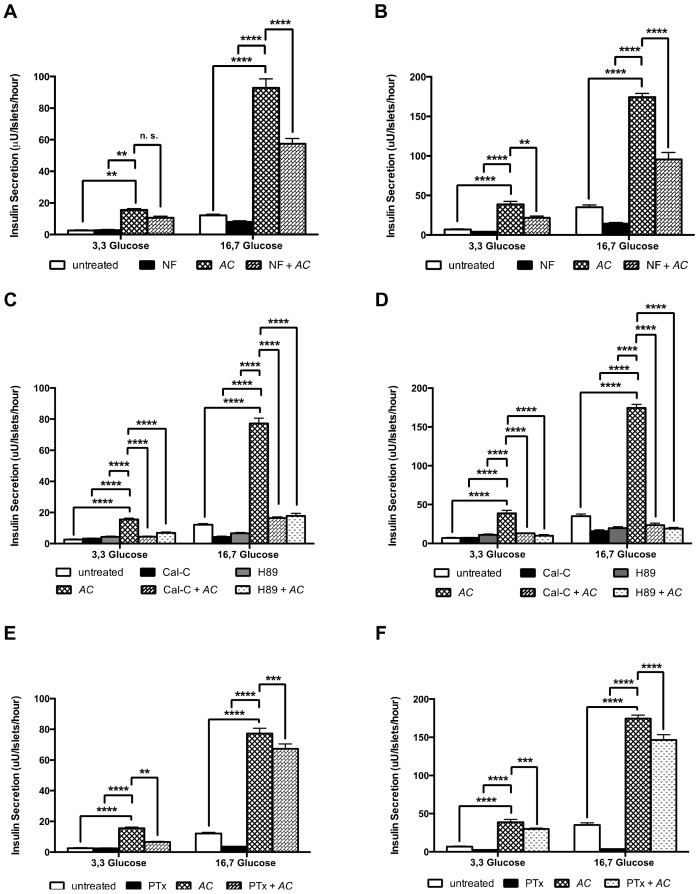
*AC* stimulates insulin secretion through PKC and PKA systems, L-type calcium channels and G protein-coupled exocytosis. The insulin secretion was evaluated in islets cultured at low (3.3 mM) and high (16.7 mM) glucose in presence of *AC* (20 mg/mL), and the different inhibitors. 10 μM NF in GK (**A**) and W rats islets (**B**); 1.5 μM Cal-C or 10 μM H89 in GK (**C**) and W rats islets (**D**); and 100 ng/mL PTx in GK (**E**) and W rats islets (**F**). Insulin concentration was measured by radioimmunoassay (RIA). Data are presented as means ± SEM (*n* = 8). ** *p* < 0.01, *** *p* < 0.001, **** *p* < 0.0001 when compared to islets treated with *AC* alone.

Nifedipine (NF), an inhibitor of L-type voltage-dependent Ca^2+^ channels, reduced the insulin secretion with 38% in GK rat islets exposed to 16.7 mM glucose (*p* < 0.001) (Figure 8A), and by 44% respectively 45% in W rat islets exposed to 3.3 mM glucose respectively to 16.7 mM glucose (*p* < 0.001) (Figure 8B).

To assess whether the *AC* effect is dependent on PKA and PKC activation, we investigated the influence of H89, an inhibitor of cyclic adenosine monophosphate (cAMP)-dependent PKA and of Cal-C, a PKC inhibitor on the *AC* modulation of insulin secretion. In GK rat islets, H89 reduced insulin secretion by 55% and by 77% at 3,3 mM and 16.7 mM glucose, respectively (*p* < 0.0001) and Cal-C reducted by 71% (3.3 mM) and by 79% (16.7 mM) (*p* < 0.0001) (Figure 8C). In W rat islets, H89 reduced insulin by 75% and by 89% at 3.3 mM and 16.7 mM glucose, respectively (*p* < 0.0001) and Cal-C reduction was 66% (3.3 mM) and 87% (16.7 mM) (*p* < 0.0001) (Figure 8D). Finally, to explore whether the *AC* effect was mediated by exocytotic G-proteins, pertussis toxin (PTx) an inhibitor of G-proteins via ADP-ribosylation was used. We found that PTx inhibited the *AC* effect in GK rat islets by 57% at 3.3 mM (*p* < 0.01) and by 20% (*p* < 0.001) at 16.7 mM glucose (Figure 8E). Similar effects were found in W rat islets, with reductions of 23% (*p* < 0.0001) and 16% (*p* < 0.001) at 3.3 mM and 16.7 mM of glucose, respectively (Figure 8F).

## 4. Discussion

One of the main features of DMT2 is the hyperglycemia that contributes to diabetes complications. Therefore, the restoration of glucose homeostasis is a main therapeutic target [7,35]. We have demonstrated that a crude extract of *AC* seeds improved glucose tolerance in the type 2 diabetic GK rats. *AC* stimulates insulin secretion, an effect mediated by PKC and PKA systems and partially by L-type calcium channel and insulin G protein-coupled exocytosis in the β-cells.

The LC-MS analysis showed that the *AC* extract consists of complex components including sugars, and minor amounts of polyphenols and amino acids. Similar composition has been described previously for *AC* and other species from the *Amaranthus* family [22]. Interestingly, polyphenols have been demonstrated to exert anti-diabetic effects [12]. Noteworthy, the glucose concentration in *AC* extract at 20 mg/mL concentration (as used in most in vitro experiments) was only 2 mM, i.e., too low to significantly enhance insulin secretion from islets exposed to 5, 10 or 20 mg/mL of the extract.

The in vivo experiments showed that a single oral administration of *AC* contributed to control the hyperglycemia in GK rats, by reducing the non-fasting glycaemia and improving the glucose tolerance through increase of the serum insulin levels. *AC* also reduced the postprandial hyperglycemia in healthy W rats by increasing serum insulin levels, however, without inducing a hypoglycemic state.

In addition, *AC* reduced the percentage of HbA1c in GK and W rats, indicating that *AC* treatment improved long-term glycemic control. Treated animals showed high serum insulin levels suggesting that the main target for the beneficial effect of *AC* is an increase of the insulin release. Indeed, the *AC* long-term treatment improved glucose-stimulated insulin secretion of pancreatic islets isolated from treated animals. The sub-acute toxicity experiments did not show any toxic effect after 28 days; thus, the treatment conditions used appear to be safe. However, further studies to test β-cell function and the impact of *AC* treatment on diabetes complications in animal models are needed.

Others have found that a methanol extract of *AC* leaves decreases blood glucose levels in rats with streptozotocin (STZ)-induced type 1 diabetes and in normal rats after seven days of treatment [25], however by an effect suggested to be exerted through inhibition of *α*-amylase [23,24]. Similar effects were reported for other species from the same *Amaranthus* genus, *Amaranthus dubius* [36], *Amaranthus Tricolour* L. [37], *Amaranthus viridis* [38,39] and *Amaranthus spinosus* Linn [40]. It is noteworthy that our investigation is the first to describe an *AC* effect on insulin secretion.

The in vitro evaluations showed that *AC* increased the insulin secretion in batch-incubated islets, an effect that was concentration-dependent and present already at rather low concentrations of the *AC* extract (lower than 20 mg/mL), for both GK and W rat islets incubated at high glucose levels. This may imply that the stimulation of insulin secretion can be achieved without or with a small risk of having hypoglycemic or toxic effects. It is also of interest that the *AC* effect on insulin secretion was comparable with the effect at the high glucose concentration of the β-cell secretagogue drug glibenclamide (Supplementary Materials Figure S5). Moreover, the *AC* effect was observed when assessing the kinetics of insulin secretion in islet perfusions. Thus, in the presence of *AC* basal insulin secretion at low glucose conditions was partially enhanced and was further stimulated by *AC* at high glucose concentrations. When *AC* was removed from the perfusion buffer, the insulin secretion returned gradually to basal levels, supporting the view that the *AC* effect is reversible and does not cause insulin leakage by any toxic effect on the β-cells. A similar effect has been described for other plant extracts and isolated compounds from natural resources [28]. In this context it is noteworthy that the *AC* extract may exert stimulatory effects on β-cell mass or islet insulin content. However, it seems unlikely that such effects are involved in its effects on insulin secretion, since the action is immediate as shown in isolated islets. Furthermore, we do not think effects on food intake are implicated, since *AC* extract treatment up to 28 days did not impact on body weight development.

When blood glucose levels are elevated after a meal, glucose is transported into the pancreatic β-cell by glucose transporter 2 (GLUT2) and metabolized via glycolysis and the Krebs cycle to produce ATP [41,42]. This results in increased cytosolic ATP/adenosine diphosphate (ADP) ratios, that leads to closure of potassium-sensitive (K-ATP) channels and further depolarization of the β-cell membrane. This in turn activates the voltage-dependent calcium (L-type Ca^2+^) channels with calcium entry into the β-cell, producing an increased cytosolic calcium concentration that stimulates insulin secretion [1,41].

To study the mechanism behind the *AC* effect on glucose-induced insulin secretion, we first evaluated the role of K-ATP channels. As expected, GK and W rat islets treated with diazoxide, which maintains K-ATP channels open [32], showed a reduction of insulin secretion. *AC* did not change the effect of diazoxide in either GK or W rat islets, suggesting that *AC* does not modulate the closure of K-ATP channels. Moreover, the *AC* effect was not altered at 16.7 glucose in GK and W rat islets depolarized by a high concentration of KCl in the presence of diazoxide. This finding suggests that the *AC* effect reached the near-maximal threshold and is not primarily dependent on membrane depolarization.

To explore the mediation of L-type Ca^2+^-channels for the effect of *AC*, we used nifedipine, an L-type Ca^2+^ channel blocker, that blocks Ca^2+^-influx into the β-cell and thereby inhibits insulin secretion [31,33]. In the presence of nifedipine, the *AC* effect at 16.7 mM glucose on GK and W rat islets was partially reduced compared to islets incubated with *AC* alone, suggesting that the *AC* effect is partially dependent on activation of L-type Ca^2+^-channels.

In addition, other intracellular signals mediated by second messengers, cAMP and diacylglycerol (DAG), can induce insulin release through phosphorylation by PKA and PKC [43]. When applying the PKA inhibitor, H89, and the PKC inhibitor, calphostin-C, the *AC* effect was significantly suppressed, in both GK and W rat islets, indicating that the *AC* effect on β-cells involves the activation of both the PKA and PKC systems. Finally, guanine nucleotide-binding proteins (G-proteins) are involved in signaling pathways of insulin secretion in pancreatic *β*-cells. Among the different G-proteins, Ge are involved in exocytosis [31,41]. In GK and W rat islets pre-treated with pertussis toxin, an inhibitor of G proteins via ADP-ribosylation, the stimulatory effect on insulin secretion by *AC* was partially reduced, indicating that the *AC* effect partially involves G protein-coupled insulin exocytosis.

Based on our findings, PKC and PKA activation appears to be important for the *AC* effect. Both enzymes phosphorylate proteins required for the initial steps of the exocytotic process [43]. One mechanism of kinase activation is mediated by Ca^2+^-activation of the receptor-coupled enzyme phospholipase C (PLC). PLC hydrolyzes the plasma membrane phospholipid phosphatidylinositol bisphosphate (PIP2) into the second messengers, diacylglycerol (DAG) and inositol trisphosphate (IP3). DAG activates PKC, and IP3 liberates Ca^2+^ from the endoplasmic reticulum [42,43]. Since an increase in cytosolic Ca^2+^ takes part in the activation of kinases, the partial inhibition of the *AC* effect by nifedipine could be explained by the lack of calcium entrance. Alternatively, the activation of adenylate cyclase by Gs protein is required for PKA activation [31,32] and PLC activation is also mediated by Gq protein [41]. Therefore, the inhibition of the *AC* effect by PTx might be explained by blocking of the synthesis of cAMP and PLC activation [41]. PKA and PKC promote insulin secretion by increasing the total number of vesicles available for insulin release and which are highly sensitive to Ca^2+^, the so called highly calcium-sensitive pool (HCSP) [43]. Thus, we hypothesize that the *AC* effect may be explained by the recruitment of more insulin granules into the HCSP through the activation of PKA and PKC.

## 5. Conclusions

We demonstrated that *AC* stimulates insulin secretion in GK and W rats by modulating steps of the glucose-dependent insulin secretion, e.g., by protein kinases A and C, effects that are dependent on intracellular increase in calcium and that partially involve the G protein-coupled exocytosis membrane proteins. The improved glucose tolerance in diabetic GK rats indicates that *AC* might be a candidate for use as a nutraceutical therapy in type 2 diabetes in man.

## Supplementary Materials

The following are available online at http://www.mdpi.com/2072-6643/10/1/94/s1, Table S1, Effect of AC long-term treatment on the OGTT test performed at day 0, day 10 and day 20 of treatment in GK and W rats, Table S2, Effect on hematological and biochemical parameters of male Wistar rats after 28 days of *AC* treatment, Figure S1, Effect on body weights of Wistar rats after 28 days of *AC* treatment, Figure S2, Effect of *AC* long-term treatment (20 days) on body weights, Figure S3, Cytotoxic effect of *AC* on W pancreatic islets, Figure S4, The *AC* effect is not mediated by ATP-dependent potassium channels, Figure S5, *AC* effect on insulin secretion was comparable with the effect of glibenclamide.
