# Supplementary material for: Amaranthus caudatus Stimulates Insulin Secretion in Goto-Kakizaki Rats, a Model of Diabetes Mellitus Type 2
Source: Nutrients. 2018 Jan 15;10(1):94. doi: 10.3390/nu10010094 (PMC5793322; doi:10.3390/nu10010094)
Supplement: Supplementary file 1 [file nutrients-10-00094-s001.pdf]

nutrients

MDPI

Supplementary Materials

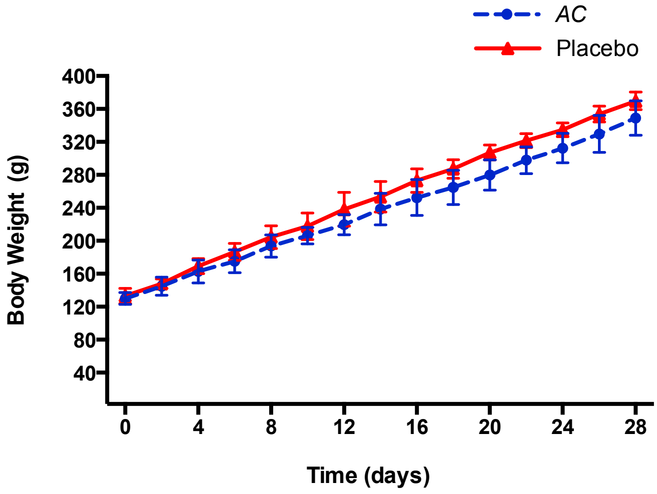

Figure S1. Effect on body weight of Wistar rats after 28 days of *Amaranthus caudatus* (*AC)* treatment. The body weight was measured every second day of treatment in W rats. Data are presented as means ± SEM (n = 6).

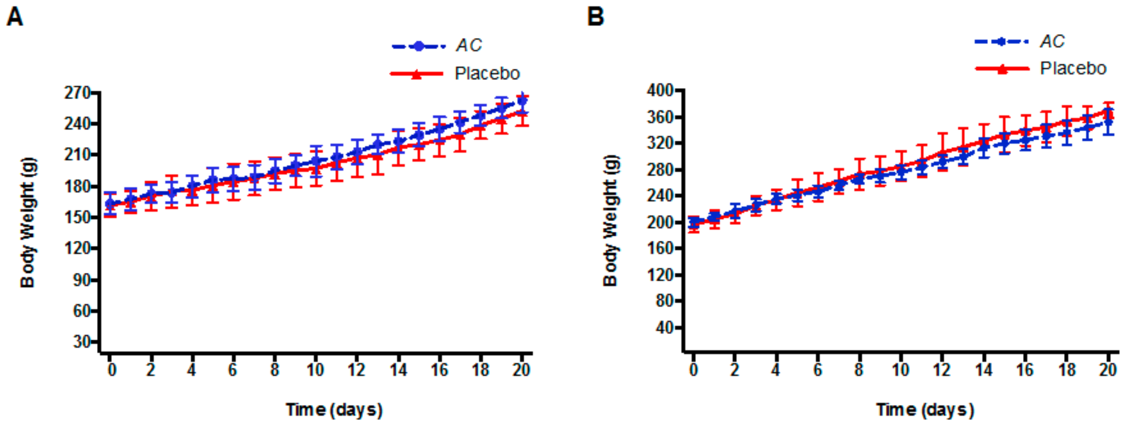

Figure S2. Effect of *AC* long-term treatment on body weight. The body weight was measured every day of treatment in GK (**A**) and W rats (**B**). Data are presented as means ± SEM (n = 6).

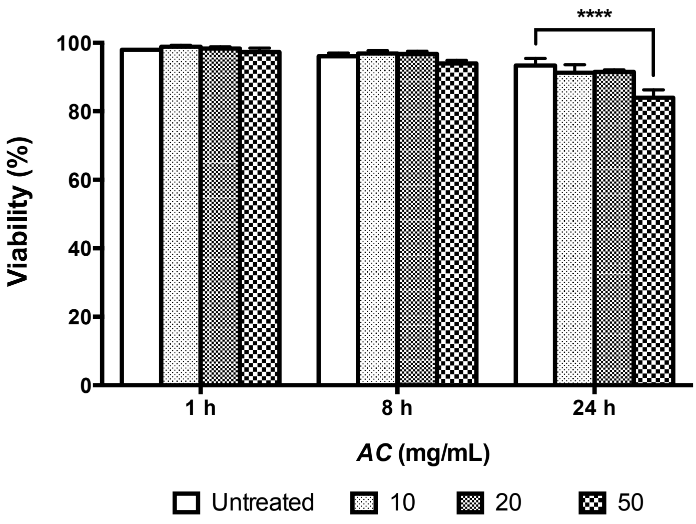

Figure S3. Cytotoxic effect of *AC* on W pancreatic islets. Panceatic islets of W rats were incubated in presence of *AC* extract (10, 20 and 50 mg/mL). Cell viability was measured by MTT assay after 1, 8 and 24h of treatment. Data are presented as means ± SEM (n = 3), of triplicates from three independent experiments.

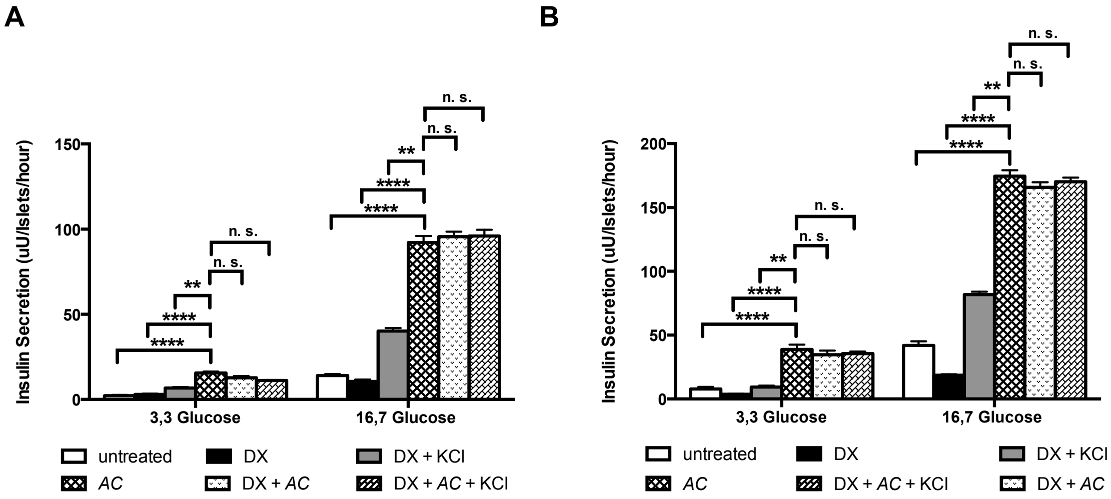

Figure S4. *AC* effect is not mediated by ATP dependent potassium channel. *AC* effect was evaluated in islets cultured at low (3.3 mM) and high (16.7 mM) glucose in presence of DX (0.25 mM) and or KCl in GK (**A**) and W rats islets (**B**). Insulin concentration was measured by RIA. Data are presented as means ± SEM (n = 8), of triplicates from four independent experiments. Data are presented as means ± SEM (n = 8). **p< 0.05, ** p<0.01, *** p<0.001, **** p<0.0001* when compared to islets treated with *AC* alone.

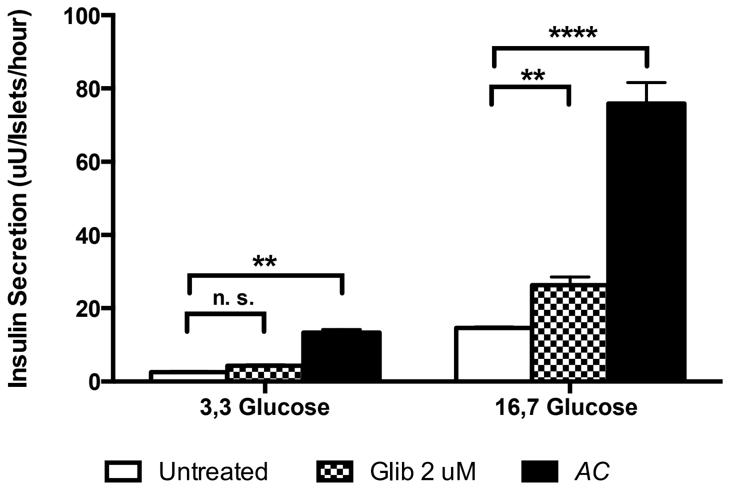

Figure S5. *AC* effect on insulin secretion was comparable with the effect of glibenclamide. Insulin secretion in GK islets was evaluated at low (3.3 mM) and high (16.7 mM) glucose in presence of a positive control Glibenclamide (2 µM) and *AC* (20 mg/mL). Insulin concentration was measured by RIA. Data are presented as means ± SEM (n = 3), of triplicates from three independent experiments.

Table S1. Effect of *AC* long-term treatment on the OGTT test performed at day 0, day 10 and day 20 of treatment in GK and W rats.

**Time (min)**	**0**	**30**	**60**	**90**	**120**
	Group 1. GK + *AC* 1000 mg/kg b.w.				
Day 0	6.3±0,1	21.1±0.3	19.7±0.3	16.0±0.5$^{\#}$	12.6±0.2
Day 10	6.6±0.2	20.6±0.6	17.6±0.2$^{***\#}$	12.8±0.3$^{****\#\#\#\#}$	10.5±0.4$^{***\#\#}$
Day 20	7.2±0.4	20.5±0.5	16.7±0.5$^{****\#\#}$	13.1±0.5$^{****\#\#\#\#}$	10.6±0.5***
	Group 2. GK + Placebo				
Day 0	7.0±0.2	21.3±0.5	19.2±0.3	17.5±0.2	13.4±0.4
Day 10	7.1±0.6	20.4±0.3	19.4±0.3	17.6±0.4	12.5±0.4
Day 20	6.9±0.2	19.9±0.3*	18.7±0.2	16.7±0.2	11.8±0.7**
	Group 3. W + *AC* 1000 mg/kg b.w.				
Day 0	4.5±0.3	11.9±0.2	10.1±0.2	7.6±0.1	6.4±0.1
Day 10	4.1±0.3	11.5±0.3	9.6±0.2	6.6±0.2***	5.6±0.2*
Day 20	4.7±0.1	9.5±0.1$^{****\#\#\#\#}$	7.9±0.2$^{****\#\#\#\#}$	6.8±0.1$^{**\#\#\#}$	5.5±0.1$^{**\#\#\#}$
	Group 4. W + Placebo				
Day 0	3.7±0.2	12.3±0.1	10.1±0.1	7.4±0.1	6.1±0.2
Day 10	4.3±0.3*	12.1±0.1	10.3±0.1	7.5±0.1	6.0±0.2
Day 20	4.6±0.3***	12.1±0.3	10.2±0.2	7.8±0.2	6.3±0.1

Data are presented as means ± SE (n = 6). **p< 0.05*, ** *p<0.01*, *** *p<0.001*, **** *p<0.0001* compared to day 0; $^{\#}$ *p<0.05*, $^{\#\#}$ *p<0.01*, $^{\#\#\#}$ *p<0.001*, $^{\#\#\#\#}$ *p<0.0001* compared to placebo group at the same time point.

Table S2. Effect on hematological and biochemical parameters of Wistar rats after 28 days of *AC* treatment

**Parameters**	**Placebo**	***AC***
**Hematological**		
Hematocrit (%)	59.8 ± 1.7	62.3 ± 2.1
Hemoglobin (g/dL)	18.1 ± 0.5	19.0 ± 0.6
Red Blood Cells (x 10^6/μL)	9.0 ± 0.5	7.0 ± 0.5
White Blood Cells (x 10^3/μL)	11.4 ± 1.4	10.7 ± 1.5
Neutrophils (%)	36.0 ± 1.6	35.5 ± 0.7
Lymphocytes (%)	64.5 ± 3.0	58.3 ± 4.1
Monocytes (%)	2.0 ± 0.3	2.3 ± 0.2
Eosinophils (%)	1.7 ± 0.2	1.3 ± 0.2
Basophils (%)	0.0 ± 0.0	0.0 ± 0.0
**Biochemical**		
Triglycerides (mg/dL)	30.0 ± 4.3	30.2 ± 5.6
Cholesterol (mg/dL)	43.3 ± 3.0	51.6 ± 3.9
Glucose (μM)	6.8 ± 0.7	5.8 ± 0.3
Creatinine (mg/dL)	0.4 ± 0.02	0.4 ± 0.02
Alkaline Phosphatase (U/L)	118.0 ± 12.6	102.0 ± 9.3
Aspartate Aminotransferase (U/L)	126.0 ± 14.6	114.6 ± 7.7
Alanine Aminotransferase (U/L)	37.0 ± 3.1	28.1 ± 3.0

Data are presented as means ± SEM (n = 6).

Raw data revealed that two animals showed higher values compared to the placebo group. Once correcting the mistake, differences between the groups were not significant. Unfortunately we do not have the basal values at the beginning of the experiment, and that information could be useful to see if those animals could have started the experiment with already high values of cholesterol. According with the literature AC and other species from the same genus have an anti-hyperlipedemic and anti-cholesterolemic effect according to the literature in STZ and aloxan diabetic models [25,39–41]. Looking at the cholesterol values of their healthy control animals, our values are not higher.

Additionally, if we review the normal values of Wistar rats from Charles River, the values of the analytes and enzymes we measured are in the range of normal values (please see the attached file). Thus, even though we observed a high mean cholesterol value in the AC group, this increase is first not significant and second it is considered in the range of normal values for healthy Wistar rats.

© 2018 by the authors. Submitted for possible open access publication under the terms and conditions of the Creative Commons Attribution (CC BY) license (http://creativecommons.org/licenses/by/4.0/).
